# A scoping review exploring oral health inequalities in India: a call for action to reform policy, practice and research

**DOI:** 10.1186/s12939-023-02056-5

**Published:** 2023-11-21

**Authors:** Parul Dasson Bajaj, Ramya Shenoy, Latha Sanjay Davda, Kundabala Mala, Gagan Bajaj, Ashwini Rao, Aparna K.S., Mithun Pai, Praveen Jodalli, Avinash B.R.

**Affiliations:** 1 Department of Public Health Dentistry, Manipal College of Dental Sciences Mangalore, Manipal Academy of Higher Education, Manipal, Karnataka 576104 India; 2 Civilian Dental Surgeon, UK and Adjunct Faculty, Manipal College of Dental Sciences Mangalore, Ministry of Defense, Manipal Academy of Higher Education, Manipal, Karnataka 576104 India; 3 Department of Conservative Dentistry and Endodontics, Manipal College of Dental Sciences Mangalore, Manipal Academy of Higher Education, Manipal, Karnataka 576104 India; 4 Department of Audiology and Speech Language Pathology, Kasturba Medical College Mangalore, Manipal Academy of Higher Education, Manipal, Karnataka 576104 India

**Keywords:** Oral health inequalities, Universal oral health coverage, Oral health policy in India, Access to dental care, Health and well-being

## Abstract

**Introduction:**

Reduction in health inequalities and providing universal access to health care have been identified as two important global milestones by the World Health Organization for countries to achieve by 2030. Therefore, recognizing the magnitude of oral health inequalities in India has become a pressing priority to improve access to dental care within the country. This scoping review was conducted with the aim of reviewing, collating and analysing the current knowledge base on oral health inequalities in India.

**Methodology:**

The scoping review followed Arksey and O’Malley’s approach, and reporting was performed in accordance with the PRISMA-ScR guidelines. A systematic search was conducted on Scopus, PubMed, Web of Science, and EMBASE to identify literature addressing one or more dimensions of oral health inequalities in India, published in English between January 2002 and April 2022. The data were charted, and qualitative analysis was performed to derive themes, highlighting the key concepts emerging from this review.

**Results:**

In accordance with the eligibility criteria, a total of 71 articles retrieved through database search and backward citation search were included in this scoping review. The major themes ranged from individual to diverse sociodemographic factors acting as barriers to and facilitators of access to dental care. Deficiencies in human resources for oral health, along with a wide diversity in dental service provision and dental education were other major themes contributing to inequality. Subsequently, this has resulted in recommendations on restructuring the dental workforce and their development and modifications in oral health care policies and practices. The qualitative synthesis demonstrates the intertwined nature of the multiple factors that influence the goal of achieving an affordable, accessible, extensive and inclusive oral healthcare system in India.

**Conclusions:**

This comprehensive review provides a broad perspective on oral health inequalities in India, providing valuable insights for both researchers and policymakers in this area and guiding their efforts towards achieving universal oral health coverage in the Indian context.

**Supplementary Information:**

The online version contains supplementary material available at 10.1186/s12939-023-02056-5.

## Introduction

Universal Health Coverage (UHC) refers to the provision of comprehensive healthcare services to every individual without encountering financial obstacles, regardless of their location or the timing of their needs. These encompass a wide array of health services in an individual’s life span ranging from health promotion and disease prevention to therapeutic care, including rehabilitation and palliative care. Among the 2030 Sustainable Development Goals (SDGs) adopted globally, achieving UHC forms a pivotal component for realizing SDG 3, which aims to ensure good health and well-being for all [1]. While there has been a growing global effort towards achieving UHC, there has been limited advancement in integrating oral health into this framework and moving towards universal oral health coverage (UOHC) [2].

Health inequalities have been recognized as the major barrier in achieving UHC by the World Health Organization [1]. Tsakos et al. referred to the term ‘health inequalities’ as ‘systematic, avoidable, unfair, and unjust differences in health outcomes,’ and throughout this paper, we will be adhering to this definition when discussing oral health inequalities [3]. Globally, inequalities specific to oral health have been established and studied extensively across various domains and contexts, encompassing high, middle and low-income countries [4–6]. In the last two decades, literature has emerged that addresses oral health inequalities in India, covering diverse areas including populations experiencing inequalities [7–13], socioeconomic factors leading to inequality [14–17], inequalities in the dental workforce [18–20] and expert opinions on addressing these gaps [21–25]. The recognition of oral health inequalities, both globally and locally, underscores the importance of comprehending the range of factors that contribute to inequalities in oral health care for effective implementation of targeted interventions.

Therefore, the first step towards attaining universal oral health coverage (UOHC) demands a thorough delineation of the various attributes that contribute to oral health inequalities. While there are existing sources of evidence that summarize various aspects of oral health inequalities worldwide [4–6], there is a lack of consolidated evidence specifically addressing this issue in the Indian context. The success of initiatives targeting oral health inequalities, as highlighted in recent literature [3], largely depends on acknowledging the context and ensuring that the proposed interventions align with the needs of stakeholders. This is crucial for effectively addressing the unique challenges associated with oral health inequalities. Although there is literature that addresses certain aspects of oral health inequalities in India, including observational studies on oral health among disadvantaged populations, it is worth noting that a comprehensive and systematically formulated body of literature specifically focused on oral health inequalities in India is currently lacking, to the best of our knowledge. Hence, this scoping review aims to provide decision-makers with comprehensive evidence base on oral health inequalities in India. By precisely documenting the contributing factors responsible for oral health inequalities in India, this review would inform policymakers and dental professionals, enabling them to channelize their efforts and resources effectively to improve access to oral health care services among the masses. Additionally, this scoping review would help to identify research gaps in the literature and delineate necessary modifications for future research in this area.

For achieving the aforementioned goals, the research question for this scoping review was defined as “What is the current evidence base on oral health inequalities in India?“ This research question was designed to fulfil three primary objectives. Firstly, to collate the extent and nature of oral health inequalities in India. Secondly, to identify the sources, types, and quality of evidence in this research area to highlight any research gaps that could guide future recommendations in addressing research related to oral health inequalities in India. Lastly, the review aimed to provide a comprehensive summary of the findings from the available literature, catering specifically to policymakers and dental professionals to facilitate informed decision-making.

## Methods

This scoping review was conducted between May 2022 and December 2022 with Arksey and O’Malley’s framework [26] as a guideline and reported in adherence with the PRISMA-ScR checklist which is provided as additional file 1 [27]. As a first step in the framework, we identified the research question with the objective of collecting data on the diverse dimensions of oral health inequalities that exist in India. Therefore, the research question was defined as “What is the current evidence base on oral health inequalities in India?“ The review protocol of the present scoping review was not registered.

### Selection criteria

Adhering to the second step of Arksey O’Malley’s framework, relevant studies were identified through a search conducted across four electronic databases (Table 1). Articles addressing one or more aspects of oral health inequalities in India that were published in English between January 2002 and April 2022 were selected. The focus was on the last 20 years, as there was a surge in the establishment of dental colleges in India and the purpose of this review was to examine the trends of oral health inequalities that followed this period of growth of dental colleges across the country. Articles where full-text formats were missing, such as conference proceedings, editorials and letters to editors, were excluded.

### Information sources and search strategy

A systematic search was performed by one author (PDB) across four electronic databases, i.e., Scopus, PubMed, Web of Science and EMBASE, from May 2022 with the last search conducted in December 2022. The search strategy, including the key terms and Booleans utilized during the initial search across various databases, is provided in Table 1.

**Table 1:** Search strategy across four databases

Database	Search String
**PubMed**	(((((((((((dental health) OR (oral health)) OR (oral health care))) AND (disparity)) OR (disparities)) OR (inequality)) OR (inequalities)) OR (inequity)) AND (india)
**EMBASE**	(((‘dental health’/exp OR ‘dental health’ OR oral) AND (‘health’/exp OR health) OR ‘oral health care’/exp OR ‘oral health care’) AND (‘disparity’/exp OR ‘disparity’) OR ‘disparities’/exp OR ‘disparities’ OR ‘inequalities’/exp OR ‘inequalities’ OR ‘inequality’/exp OR inequality OR inequity) AND (‘india’/exp OR ‘india’) AND [english]/lim AND ([embase]/lim OR [medline]/lim)
**Web of Science**	((((((((ALL=(oral health)) OR ALL=(dental health)) OR ALL=(oral health care)) AND ALL=(disparity)) OR ALL=(disparities)) OR ALL=(inequality)) OR ALL=(inequalities)) OR ALL=(inequity)) AND ALL=(India) and Public Environmental Occupational Health or Multidisciplinary Sciences or Environmental Sciences or Infectious Diseases or Surgery or Medical Informatics or Education Scientific Disciplines or Medicine General Internal or Health Policy Services or Social Sciences Biomedical or Health Care Sciences Services or Geography or Demography or Women S Studies or Area Studies or Pediatrics or Social Issues or Ethnic Studies or Cultural Studies or Pathology or Primary Health Care (Web of Science Categories) and English (Languages)
**Scopus**	( ALL ( oral AND health OR dental AND health OR oral AND health AND care ) AND ALL ( disparity OR disparities OR inequality OR inequalities OR inequity ) AND ALL ( india ) ) AND PUBYEAR > 2001 AND PUBYEAR < 2023 AND ( LIMIT-TO ( LANGUAGE , “English” ) )

### Study selection

The initial search results across the four databases were screened for titles by one author (PDB), which yielded 206 articles. Adhering to the selection criteria described above, these articles were subjected to title and abstract screening followed by full-text review by two authors (PDB and RS) independently. Any disagreements were resolved by discussion with the help of a third author (LD). According to the eligibility criteria, a total of 53 articles were selected following full-text review. One author (PDB) conducted a backward citation search on the references of the chosen articles, resulting in the identification of 54 records, which again underwent title and abstract screening followed by full-text review by two authors (PDB and RS) independently.

### Charting the data

The data from the selected full-text articles for the current scoping review were independently charted by two authors (PDB and AKS) using a pre-designed format in Microsoft Excel software version 16.54. This format facilitated the collection of information such as author(s), year of publication, study location, type of study/article, study population, aim of the study, methods used, outcome measures, and significant findings. The data extraction tables were later collaboratively reviewed to identify and complete any missing information that was pertinent to the review and combined to give a more comprehensive picture presented by the chosen articles.

### Critical appraisal of the selected articles

This scoping review was undertaken primarily to explore and analyse the existing evidence on oral health inequalities in India, including the types of evidence available, and to identify any gaps in the literature. Therefore, a quality appraisal of the selected sources of evidence was undertaken to accurately determine the research gaps within the current body of literature. The Crowe Critical Appraisal Tool (CCAT) facilitates the critical evaluation of papers covering a diverse array of research designs [28]. Hence, for the current scoping review, we opted to use CCAT because there were no limitations on study designs during the article selection process for this review. The quality appraisal of the selected articles was performed by two authors (PDB and GB) independently. The CCAT evaluates each paper across eight aspects: preliminaries, introduction, design, sampling, data collection, ethical issues, results/findings, and discussion. Each category is scored individually on a scale of 0–5, resulting in a maximum potential score of 40. In case of any conflicting ratings, both authors engaged in a collaborative discussion with a third author (LD) to reach a consensus. For ensuring accuracy, a few articles were randomly selected and subjected to quality appraisal by the third author (LD). Based on the appraisal scores, papers receiving a total score of 35 or higher were classified as high quality, those scoring between 25 and 34 were considered medium quality, and papers scoring below 25 were deemed low quality.

### Synthesis of results

Following the last step of Arksey O’Malley’s framework, which involves collating, summarizing, and reporting results, each selected article was subjected to qualitative thematic analysis. One author (PDB) extensively reviewed the extracted data from the selected articles, comparing them with the corresponding full-text articles. Through an open coding process, relevant sections of the full-text articles that addressed the research question on oral health inequalities in India were identified and labelled as initial codes, utilizing ATLAS.ti Mac (Version 23.2.0). At this stage, a second author (RS) was invited to independently review the initial codes. Subsequently, both authors (PDB and RS) integrated and refined the initial codes to arrive at the final codes. These final codes underwent further integration, refinement, and categorization to generate sub-themes aligned with the objectives of the study with assistance from the third author (LD). In the subsequent step, the sub-themes were organized to identify and establish main themes based on their core concepts by two authors (PDB and LD). Through comprehensive discussions, the three authors (PDB, RS, and LD) thoroughly examined and assigned labels to the main themes, followed by tabulation of the main themes and sub-themes to enhance clarity and facilitate understanding.

## Results

The results are presented as a summary of the sources, types, and quality of evidence followed by the main themes and subthemes regarding inequalities in oral health in India.

### Sources, types, and quality of evidence

**Fig. 1:**
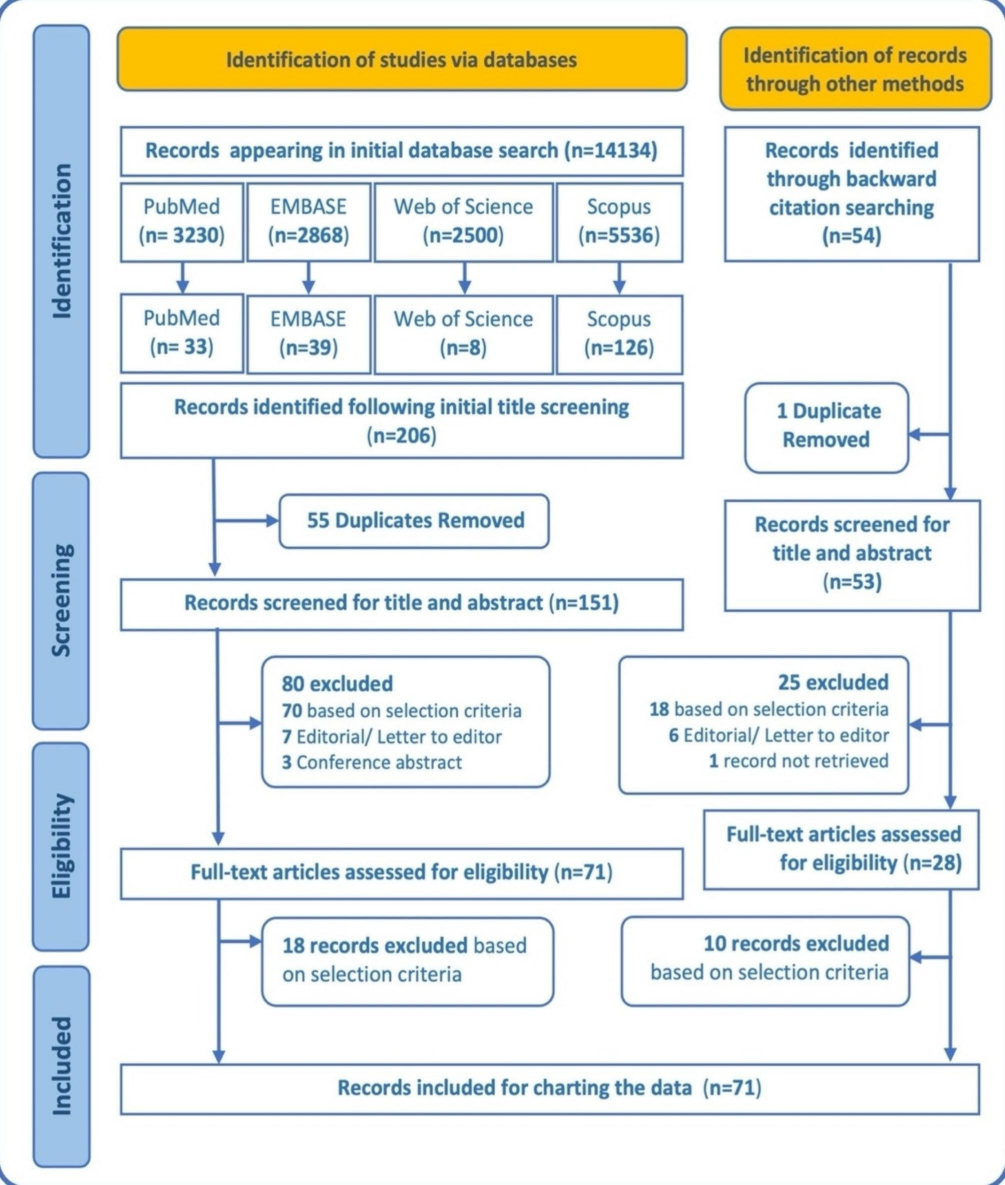
PRISMA flow diagram [29] depicting the process of article selection for the scoping review

Based on the eligibility criteria, a total of 71 articles retrieved via database search and backward citation search were included in this scoping review. Figure 1 displays the PRISMA flow diagram for this scoping review [29], illustrating the details of the selection process from initial search to final inclusion.

**Fig. 2:**
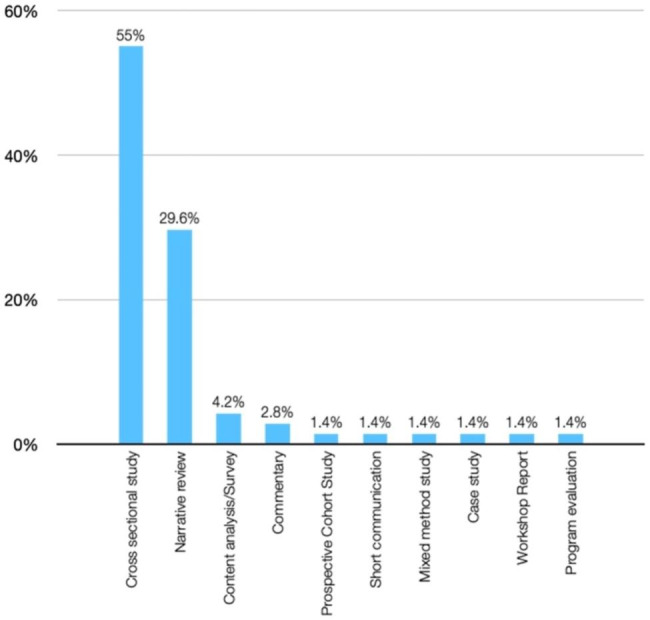
Distribution of selected sources of evidence according to type

**Fig. 3:**
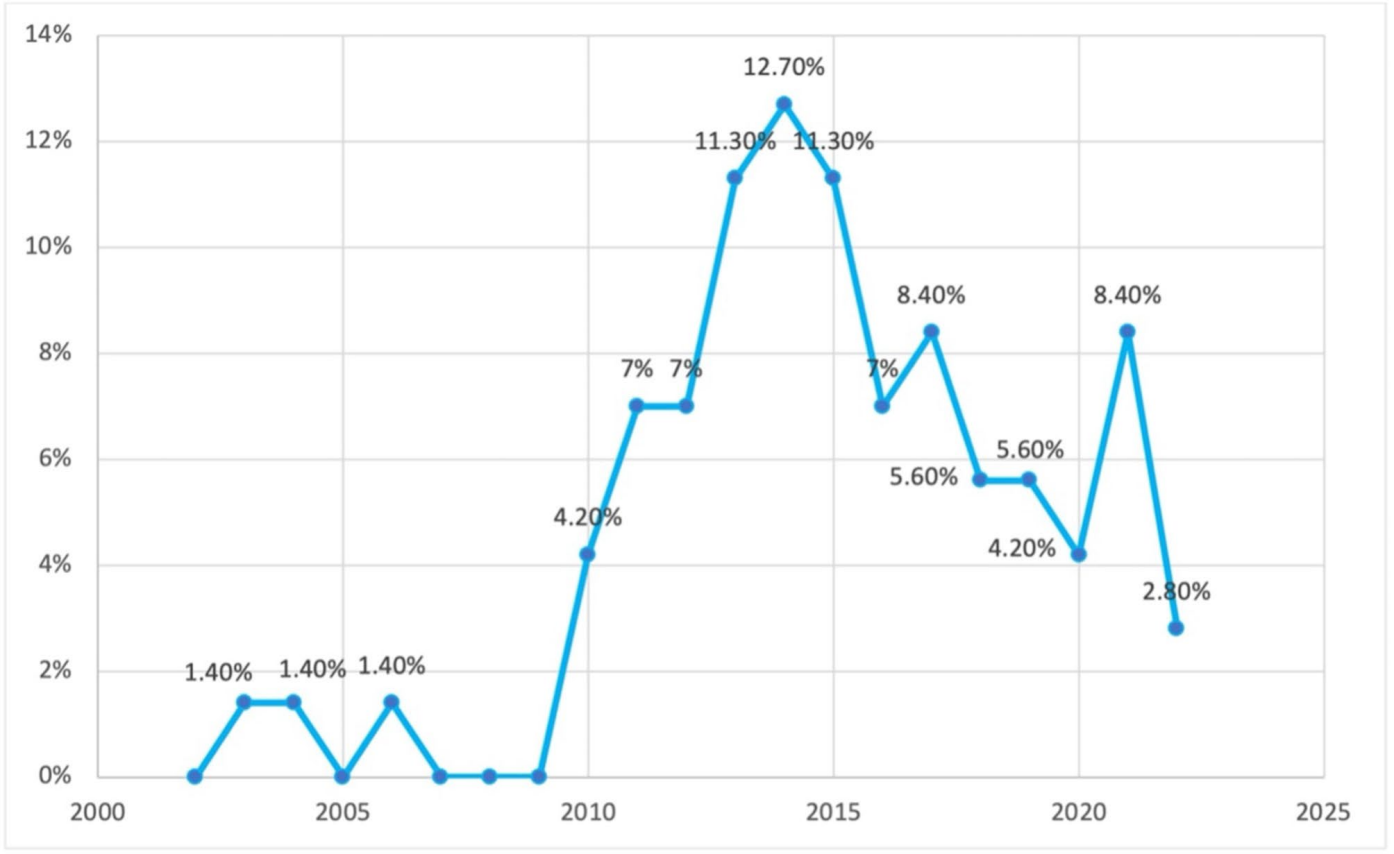
Distribution of selected sources based on year of publication

Among the sources analysed, a significant portion (55%) were cross-sectional studies. Additional file 2 in the supplementary materials provides a detailed presentation of the features of the evidence sources selected for this scoping review, as well as the data that have been charted from these sources. In Fig. 2, the distribution of the 71 selected papers is illustrated based on the type of study. The majority of the papers were cross-sectional studies (55%), followed by narrative reviews (29.6%). Figure 3 demonstrates that the papers included in this scoping review were predominantly published in the last decade highlighting an increase in research in this area.

The quality appraisal of the selected sources of evidence was performed using CCAT, and detailed scores for each paper are presented in Additional file 3 in the supplementary material. According to the criteria devised by the authors based on the total CCAT scores, the papers were labelled high, medium and low quality for ease of understanding. Among the 71 papers selected for this scoping review, 8 were high-quality papers (11.3%), 42 were medium-quality (59.1%), and 21 were low-quality (29.6%). Most of the cross-sectional studies were evaluated to be of medium quality (84.6%), while few were high quality (12.8%). The majority of the narrative reviews, accounting for 29.6% of the total papers, were deemed to be of low quality (85.7%) due to a lack of methodological clarity. The segregated classification based on quality for the selected papers across various study designs is depicted in Table 2.

**Table 2:** Quality assessment of selected papers across various study designs

Type of Study Design	High Quality Percentage (number)	Medium Quality Percentage (number)	Low Quality Percentage (number)
Cross sectional study	12.8% (5)	84.6% (33)	2.6% (1)
Narrative review	4.8% (1)	9.5% (2)	85.7% (18)
Content analysis/Survey	**-**	100% (3)	**-**
Commentary	**-**	50% (1)	50% (1)
Prospective Cohort Study	100% (1)	**-**	**-**
Short communication	**-**	100% (1)	**-**
Mixed method study	100% (1)	**-**	**-**
Case study	**-**	100% (1)	**-**
Workshop Report	**-**	**-**	100% (1)*
Program evaluation	**-**	100% (1)	**-**

*- This paper was included due to its focus on health inequalities in a specific disadvantaged group and presented practical approaches for driving policies

Quality assessment was performed using the Crowe Critical Appraisal Tool [28].

### Main themes and subthemes concerning oral health inequalities in India: Breaking down the complexities, policy implications and solutions

Each of the main themes is reported with its key findings as listed in Table 3, along with the reference papers. The main themes and sub-themes were organized in a hierarchical manner, starting with individual factors, followed by socio-demographic characteristics and factors influencing access to dental care. This is followed by operational factors influencing oral health care, beginning with the current deficiencies in dental services, dental workforce development and policies, followed by the proposed solutions for each of these areas.

**Table 3:** Main themes and sub-themes regarding inequalities in oral health in India

Main Themes	Sub-themes	References
**Individual Factors**	Fear & anxiety	[2, 7, 8, 11, 13, 40, 45, 52, 55, 75–80]
	Cultural and personal beliefs	[2, 7, 8, 11, 17, 21, 22, 25, 30, 31, 34, 37, 40, 42, 52, 55, 57, 63–65, 67, 69, 73, 75–81]
	Preference for alternative medicine	[8, 13, 25, 37, 55, 80]
	Time pressures	[11, 40, 52, 55, 57, 76, 77, 80]
	Lack of awareness	[2, 7, 11, 17, 19, 22, 24, 25, 31, 36, 37, 40–44, 51–53, 57, 63–67, 70, 73, 75–82]
	Higher risk for oral cancer and periodontal disease	[21, 68, 83]
	Patient Perception	[57]
	Misc. Personal Barriers	[11, 31, 45, 55, 69, 70, 77]
**Socio-demographic Factors**	Age [Infants (0–5 yrs.),Children (6–18 yrs.), Adults (Above 18 yrs.), Geriatric Patients]	[12–14, 16, 25, 30, 36, 38, 43, 55, 66, 67, 73, 81]
	Gender (Females, Transgender’s)	[7, 13, 14, 30, 31, 55, 68, 72, 74, 77, 79, 84]
	Geographic Distribution (Rural, Urban)	[14, 16, 19, 21, 30, 31, 39, 44, 55, 57, 62, 64, 66, 68, 73, 81]
	Underserved Areas (Resettlement Colonies, Urban Slums, Tribal People, Immigrants, Socially isolated)	[12, 14, 16, 21, 36, 40, 44, 54, 63, 64, 66, 69, 76, 80, 81]
	Disadvantaged High Needs Group	[8–10, 21, 45, 71, 72, 82]
	Socioeconomic Factors	[10, 14–17, 21, 30, 31, 44, 55, 62, 64–66, 68, 71, 78–82]
	Deprivation	[14]
**Access Barriers & Facilitators**	Inability to travel	[40, 63, 66, 76, 77, 81]
	Unable to afford the cost of care	[2, 7, 17, 19, 21, 24, 25, 34, 36, 39, 40, 43, 44, 46, 52, 53, 55, 57, 67, 76, 77, 80]
	Lack of public dental care	[8, 13, 43, 50, 63, 84]
	High cost of private dental care	[19, 24, 30, 39, 46, 48, 51, 53, 65, 67, 85]
	Facilitators of oral health	[13–15, 17, 22, 30, 31, 55, 61–66, 68, 73, 79, 81, 82]
**Deficiencies in Human Resources for Oral Health**	Dental education and training	[11, 18, 21, 23, 24, 31, 36, 39, 43, 53, 54, 70, 86]
	Human Resource planning and distribution	[2, 18–21, 23, 24, 31, 34, 36–39, 41, 43, 44, 46, 50, 52, 53, 55, 74]
	Under and non-utilization of dental auxiliaries	[20, 23, 24, 34, 37, 39, 43, 48, 52, 86]
**Shortcomings of Dental Service Provisions**	Limitations of Public set-ups	[2, 11, 18, 19, 21, 23, 24, 36, 37, 39–43, 46, 52, 55–57, 67, 77, 78, 82]
	Dentist related factors	[7, 8, 40, 52, 55, 57, 70]
	Policy related issues	[2, 18, 19, 21, 23–25, 34, 36–39, 43, 46, 48, 50–53, 55, 80]
**Restructuring of dental workforce development strategies**	Dental education and training reforms	[7, 11, 12, 15, 18, 19, 23–25, 31, 32, 34, 36, 38, 41–45, 48, 50, 51, 55, 56, 66, 70, 72, 74, 86]
	Dental workforce related reforms	[18–20, 22–25, 30, 31, 36–39, 42–44, 48, 51, 52, 54, 74, 86]
**Modifications in oral health policies and practices**	Public Health related reforms	[2, 8, 11, 12, 14, 15, 18, 20–25, 30–32, 34, 36, 37, 41–46, 48, 51–53, 62, 66–68, 73, 74, 82, 83, 86]
	Oral Health Promotion related reforms	[2, 11, 12, 14–17, 21–24, 30, 32, 34, 36, 38, 40–45, 48, 50–52, 55, 56, 62, 65, 67–70, 73, 77, 82, 83]
	Policy related reforms	[2, 8, 11, 12, 15, 18–22, 25, 30, 31, 34, 36–44, 46–48, 50, 51, 53, 55, 57, 62, 63, 65, 74, 84]
	Infrastructure Reforms	[2, 19, 21, 22, 24, 34, 36–38, 40, 42, 44, 48, 50, 52, 56]

*Individual factors* were associated with how the knowledge, behaviours, beliefs, and perceptions of the studied populations influence their utilization of accessible dental care services. For instance, fear at different levels, such as fear of dental treatment, discrimination, or concerns about being financially taken advantage of by dental professionals, influenced individuals and deterred them from seeking necessary dental treatment.

*Socio-demographic factors*, such as age, gender, geographical location, and socioeconomic factors, encompass a range of elements that contribute to the decision-making process of individuals when accessing dental care services. Both individual and socio-demographic factors can directly or indirectly give shape to the *emerging barriers and facilitators in accessing oral health care services*, which further include factors like affordability, cost of care and other factors such as transportation.

*Deficiencies in human resources in oral healthcare* emerged as an important theme centred on organizational aspects. For example, when examining factors relating to human resources for oral health, the thematic analysis revealed shortcomings in dental education, inadequacies in dental workforce planning and the limited utilization of dental auxiliaries. Interestingly, several solutions were also suggested in the selected papers, forming another main theme of *restructuring of the dental workforce and their development strategies.*

Furthermore, at the organizational level, the next main theme was the *shortcomings in the provision of dental services*, with primary emphasis on identifying deficiencies in current policies and recognizing constraints within public health systems. In conjunction with this, a diverse range of strategies were proposed to enhance oral healthcare services and policies in India. These measures constituted the last main theme of *modifications in oral health policies and practices*, which included promoting oral health, implementing policy reforms, improving public health systems, and upgrading infrastructure. In the next section, these above results are discussed in the context of current literature, along with their relevance to oral health care in India, followed by seven key recommendations.

## Discussion

The purpose of this scoping review was to conduct a comprehensive examination of oral health inequalities in the Indian context. The goal was to establish a solid groundwork for guiding policy makers and dental professionals in addressing these issues and unearthing the research gaps in this area by examining the currently available evidence.

### Summary and interpretation of the results

A total of 71 papers were considered for the synthesis of evidence in this scoping review. Most of the papers included in this scoping review were published within the last decade, highlighting the significance and urgency of addressing oral health inequalities in India. The main themes were organized in a structured manner, progressing from individual factors to intermediate sociodemographic factors and their influence on access to care. Following that the themes encompassed structural and organizational factors, such as dental workforce, their education and public and oral health policies that contribute to oral health inequalities. This approach aligns with recent literature, and therefore provides insights into the theoretical frameworks and research pathways that should be considered when conducting research on oral health inequalities [3]. While the main themes follow this arrangement, it is important to note that the core concepts emerging from different themes are intricately linked and exert influence on each another. Therefore, in the subsequent five sub-sections, the key principles derived from the themes at various levels within this hierarchical order will be explored together, interweaving with one another to provide a deeper understanding, rather than examining them individually.

### Oral health inequalities in India: upstream and downstream

In the realm of oral healthcare policy, there is a growing recognition to transition from traditional behavioural downstream approaches towards more comprehensive upstream approaches to counter oral health inequalities [8, 31, 32]. The upstream approaches will ensure a more sustainable improvement in oral health promotion since these focus on creating a social environment that is conducive to good oral health for the entire population through fiscal measures, legislation, and local and national policy initiatives [33]. This complements efforts to combat the lack of awareness of the importance of oral health, a significant individual factor identified in this review, which influences how people perceive oral health and ultimately their utilization of oral healthcare services. While upstream strategies are crucial, it’s essential to acknowledge the continued importance of downstream approaches in the Indian context. Individual factors like fear, cultural beliefs, and patient perceptions significantly impact oral health and service utilization. Therefore, in alignment with the recommendations presented by Watt [33] and supported by evidence from various sources in this scoping review [8, 34], adopting complementary strategies that encompass both downstream and upstream approaches in oral healthcare within the Indian context could be a promising path towards achieving oral health equality.

### Oral health inequalities in India: harnessing new and leveraging existing systems

Estimates indicate that the treatment of oral diseases is the fourth most expensive in developed nations and can surpass the entire healthcare budget assigned in several countries [35]. This partly explains the insufficient funding dedicated to oral healthcare in India, as emphasized by the findings of this review [2, 21, 36–38, 85], thereby highlighting the need to prioritize oral disease prevention over treatment. The review reveals a contrasting situation in the Indian oral public health sector, where there is a lack of emphasis on organized primary oral health programs, with greater attention given to tertiary treatments in government healthcare packages [2, 21, 39].

Consolidating the diverse range of recommendations analysed in this review, it is recommended that instead of generating additional resources for oral health, a more effective approach would be to optimize the utility of the available limited public health resources available. This can be achieved by prioritizing primary prevention and health promotion, utilizing the existing healthcare systems in India. In light of this, the common risk factor approach, which integrates general and oral health, is crucial [12, 15, 20, 22, 37, 38, 40, 41]. Notably, it has been observed that 47.4% of the urban population seeks oral and maxillofacial care at Primary Health Centers (PHCs) maintained by the state government, where non-dental primary care providers, such as medical officers at PHCs, serve as the initial point of contact [42]. Consequently, it becomes crucial to equip all medical professionals and healthcare workers with essential oral healthcare knowledge as well as integrate oral health and medical services at various levels ranging from providing referrals and utilizing technology for virtual integration to sharing locations and funds and ultimately achieving complete integration [22, 36, 42–44].

Available resources could be channelized by utilizing the services of Accredited Social Health Activists (ASHAs) and Anganwadi workers for oral health promotion, particularly in rural areas [45]. Furthermore, a significant number of papers emphasized the need for partnerships encompassing public‒private collaborations, collaborations across various health professions in both public and private sectors, partnerships between the government, private sector, and civil society, and community and public partnerships [21, 25, 31, 32, 34, 37, 41, 42, 44, 46–48]. More specifically, allocating funding for community preventive oral health programs conducted by dental colleges in India, implementing community-based oral health programs specifically targeting government-run schools in rural areas and forming partnerships with charitable organizations that are well received by the community, are a few examples in this realm.

An imperative element of optimizing existing resources involves embracing successful healthcare models tailored to oral health promotion within the Indian context. For example, the “Fit for School” program employed for school children in the Philippines, where handwashing, periodic deworming was included with toothbrushing with fluoridated toothpaste [49]. This initiative employs a directed population approach and has the potential to be replicated in India, with a particular emphasis on children in rural areas, providing a sustainable and cost-efficient method to promote both oral and general health on a large scale. Furthermore, established frameworks of successful public health campaigns, such as the “Pulse Polio” and the “Mid-Day Meals Scheme,“ can be utilized to enhance oral health initiatives for children [32].

Adoption of novel approaches is equally important to enhance the existing system, like the implementation of updated oral health care policies with necessary modifications [18, 19, 30, 31, 38–40, 43, 50], having adequate representation of dentists in decision-making bodies [18, 23, 51], allocation of sufficient resources to establish dedicated dental units within Primary Health Centres, employing cost-effective measures such as utilization of dental auxiliaries in primary healthcare and embracing newer technological advancements, such as teledentistry for remote dental consultations and leveraging technology for oral health promotion [44, 50, 52].

### Oral health inequalities in India: demand vs. supply

Access to oral health care is primarily influenced by demand and supply dynamics [21]. The supply side of this equation, as revealed by this scoping review revealed significant inadequacies in the development of human resources for oral health in India, particularly an uneven distribution of dental colleges across rural and urban areas, as well as among different states [18, 21, 23, 24, 31, 39, 53, 54]. This disparity is further magnified with shortage of dental professionals working in rural areas and limited opportunities in the public health sector, resulting in an imbalance in the allocation of dentists, between the private and public sectors as well as between urban and rural regions [18–21, 23, 24, 37–39, 41, 43, 44, 46, 53, 55]. It has been found that public dental facilities, like Primary and Community Health Centres, face shortages of dental professionals, including dentists and dental auxiliaries, and the available dentists are underutilized due to a lack of necessary equipment and materials [2, 21, 23, 37, 42, 46, 52, 56, 57].

In alignment with the World Health Organization’s vision document “Global strategy on human resources for health: Workforce 2030” [58], ensuring universal access to oral healthcare in India requires strategic planning to balance the distribution of dental colleges and optimize the utilization of existing dental graduates to reshape the dentist-to-population ratio in underserved areas. Promoting rural dental practices, encouraging dentists to work in the public sector and primary care, and increasing the use of skill-mix are essential to ensure better dental workforce planning. This would also contribute to tackling the issue of Indian dental graduates leaving the country due to factors such as shortcomings in the oral healthcare system, a scarcity of public sector job opportunities, and career stagnation [87]. Better utilization of dental auxiliaries within the Primary Health centres could be more cost-effective and support dentists with high workload [57]. Global trends suggest that dental auxiliaries have the potential to play a crucial role in oral disease prevention, health promotion, raising awareness, and improving access to oral care services. Skill mix models are utilized across the globe, where the services of dental therapists, dental hygienists and dental nurses are employed to enhance access to oral health care [59, 60, 88–90].

Directing attention to the demand side of this dynamic, individual factors such as patient perception, perceived needs, and cultural beliefs play a vital role in determining the utilization of dental services [21]. Therefore, consideration should be given to enriching the population’s oral health literacy and modifying health behaviours to enhance the utilization of available dental services.

### Oral health inequalities in India: accessibility and affordability

Accessibility and affordability significantly affect healthcare utilization [42]. Based on the results of this scoping review, access to oral healthcare is influenced by socio-demographic factors like education, income, social support, and area of residence, particularly in rural areas, urban slums and resettlement colonies [13–16, 19, 22, 30, 31, 55, 61–68]. Recommendations to improve access to dental care include a primary healthcare approach and community-centered dental education, which involve measures like increasing the number of public health dentists, implementing mandatory rotations in mobile dental vans, satellite clinics, and rural areas, as well as establishing partnerships between dental colleges and Primary Health Centers (PHCs) and districts.

Affordability emerged as a crucial factor in this scoping review. Some of the barriers to accessing dental care included financial constraints, such as the inability to afford the cost of care, high expenses in the private sector, the absence of dental insurance plans in India as well as transportation difficulties and the associated costs of travelling. This can be better understood through a statement made by Budetti et al., where they described people who could not afford health care as “people who cannot afford to get sick” [21].Therefore, policy and infrastructure reforms involving measures such as providing dental insurance, subsidizing dental care and products, promoting local production of dental materials to reduce costs, and lowering taxes on dental health products such as toothpaste emerged as important avenues to enhance the affordability of dental healthcare among the masses.

### Oral health inequities in India: extensive and inclusive

An extensive understanding of the socio-demographic factors brings us closer to grasping the contextual landscape from which we must operate when addressing inequalities in oral health care in India. Therefore, the implementation of organized data recording systems to maintain electronic dental health records in the Indian context becomes significant [36, 38, 52] to better understand the dental needs of various stakeholders belonging to different communities and populations. Inequalities can be based on gender, age, area of residence, social position, and particularly among marginalized populations, including socially isolated individuals such as tribal people [12], immigrants [69], as well as the disadvantaged high-needs group, which comprises individuals with special care requirements [10, 45, 70], health conditions such as HIV leading to discrimination [7, 8], visually impaired individuals [71], orphans [9], and patients with substance abuse issues [72].

Oral health care services can be made more inclusive by tailoring educational programs to meet the needs of various stakeholders, including diverse ethnic groups and underprivileged populations [12, 30, 44, 65, 69], as well as implementing school-based oral health programs [55, 67, 73], initiatives in old-age homes, institutionalized settings as well as implementing community outreach programs for individuals affected by HIV [8]. These proposals align with the directed downstream population approach, which targets specific populations within specific geographic areas [32]. Moreover, these proposals encompassed replicating existing dental outreach models to reach inaccessible populations and utilizing mobile dental units to provide educational initiatives in underserved areas [12, 32, 44].

Enhancing the dental curriculum with a focus on behavioural sciences, ethics, and social responsibility is vital for preparing future dental professionals to serve underprivileged and disadvantaged groups effectively [11, 12, 23, 38, 48, 55, 70, 74]. These measures aim to build empathy among trainees and promote inclusiveness in oral healthcare, ultimately creating an extensive and inclusive oral healthcare delivery system.

### Implications of the review

Overall, the findings of this scoping review indicate that we have a substantial, albeit fragmented, understanding of the extent and magnitude of oral health inequalities in India. It seems crucial to move beyond mere documentation and translate this knowledge into practical applications in the field of oral healthcare in India. Policy interventions need to be built upon solid foundations that intricately weave together various factors in the hierarchical framework while recognizing the interdependency among these factors to achieve significant advancements and promote equality in oral healthcare.

Seven key recommendations are proposed as a result of this comprehensive scoping review. First, implement measures that promote the integration of general and oral health across various domains. Second, educate all medical and healthcare workers with essential oral health care knowledge, while also increasing awareness among the population about the significance of oral health and its connection to general well-being. One potential avenue is to propose that health profession councils introduce ongoing professional development courses specifically focused on oral health. Third, fostering collaborations between private and public entities, across diverse health disciplines, communities, and organizations, to enhance access to oral care. Fourth, a crucial practical recommendation is to establish dedicated dental units within Primary Health Centres, equipped with the necessary dental materials, instruments, and workforce to ensure effective operation. The fifth recommendation is to make oral disease prevention cost effective through the utilization of dental auxiliaries within primary healthcare frameworks. The sixth recommendation calls for a shift in dental education toward a more community-oriented approach to sensitize the upcoming dental graduates towards the needs of the community. Lastly, to remain attuned to the prevailing realities of the population, the seventh recommendation suggests implementing organized data recording systems for maintaining electronic dental health records to closely monitor the oral healthcare needs of the population.

### Strengths and limitations

This scoping review has provided an in-depth analysis of the literature on oral health inequalities in India through qualitative synthesis of data, which is unique. One of the limitations is that the sources of evidence were confined to four electronic databases, however they were sufficient to identify the existing evidence base. Additionally, some articles that received low-quality scores during critical appraisal were included in the analysis. This decision was made to ensure the inclusion of all relevant literature, aiming to offer a more comprehensive overview of oral health inequalities in India. Although the research designs of the papers included were varied, utilising a structured framework enabled identification of the complex interlinkages and influences they exerted on the oral health system in India.

### Future research directions

A need for more higher-quality observational studies and a shift from narrative to more systematic and meta-analytical reviews was identified as essential in establishing robust evidence concerning oral health inequalities in India. Additionally, it is also important to highlight that only one mixed-methods study was found [11], which thoroughly explored the issue of oral health inequality using qualitative methods. Future research in this area, particularly when studying disadvantaged populations, should also prioritize the inclusion of stakeholders’ perspectives and individual’s lived experiences as a central component [3]. This can be achieved by employing qualitative research methods in future investigations to gain a deeper understanding of the underlying causes of oral health inequalities.

## Conclusion

This comprehensive review provides a broad perspective on oral health inequalities in India, providing valuable insights for both researchers and policymakers in this area and guiding their efforts towards achieving universal oral health coverage in the Indian context. The spectrum of factors contributing to these inequalities encompass individual and sociodemographic aspects, access considerations, human resources for oral health, various dimensions of dental services, and policy considerations. The qualitative synthesis unveiled the complexity and the intricate interplay among these elements to progress towards universal oral health coverage. The review has identified a lack of awareness among the population and health care providers regarding the importance of oral healthcare and its connection to overall health. Furthermore, it has highlighted limited public funding, necessitating a redirection of resources towards primary prevention strategies rather than solely focusing on treatment of disease. It is imperative to enhance the integration of oral and general healthcare through partnerships involving public, private, and educational institutions. Additionally, there is a pressing need to create and support a well-trained dental workforce, which should include skilled dental auxiliaries. The review culminates in seven recommendations that underscore the effectiveness of employing complementary approaches in oral health, restructuring the dental workforce, and integrating new strategies while optimizing existing systems. These measures aim to address the need for an affordable, accessible, extensive, and inclusive oral healthcare system in India. Furthermore, the review emphasized the necessity for higher-quality experimental research that incorporates qualitative research methods. Overall, this scoping review provides a panoramic view of oral health inequalities in India, to inform policymakers and ensure oral healthcare for all.

### Electronic supplementary material

Below is the link to the electronic supplementary material.

Supplementary Material 1

Supplementary Material 2

Supplementary Material 3

## Data Availability

The data that supports the findings of this study is available within the manuscript and no additional source of data is required.
