# Supplementary material for: A scoping review exploring oral health inequalities in India: a call for action to reform policy, practice and research
Source: Int J Equity Health. 2023 Nov 21;22:242. doi: 10.1186/s12939-023-02056-5 (PMC10664303; doi:10.1186/s12939-023-02056-5)
Supplement: Supplementary file 2 — Supplementary Material 2 [file 12939_2023_2056_MOESM2_ESM.docx]

| **Author(s), year of publication, study location** | **Type of Article** | **Study Population** | **Aim of Study** | **Methodology** | **Outcome Measures** | **Important Results** |
| --- | --- | --- | --- | --- | --- | --- |
| Kailembo et al, 2018, Secondary data  from China, Ghana and India | Cross-sectional survey | Subjects were adults aged 50 years or more from Ghana, China and India | Measurement and description of  self-reported oral health unmet needs with respect to education related and socioeconomic inequalities | Data from the WHO SAGE Wave 1 (2007–2010) . Unmet needs were measured and compared with respect to education and household wealth. | Unmet needs [measured in terms  of Relative index of inequality (RII) | In India, prevalence of unmet needs in adults older than 50 years with respect to oral health was 62%, wherein it was more in adults who were from rural areas, were less educated and not working. |
| Mathur et al, 2016, Delhi | Cross-sectional study | Adolescents between the age of 12-15 years | To assess the socioeconomic  inequalities and determinants in oral hygiene | Three groups segregated on the basis of socioeconomic status in accordance with area  of residence from urban slums, middle-class areas and resettlement colonies. Simplified Oral Hygiene Index (OHI-S) was used to examine oral hygiene levels. | Oral hygiene status | Majority had poor oral hygiene.  Adolescents from urban slums and resettlement colonies had higher numbers of having poor oral hygiene too. |
| Mathur et al, 2014, New Delhi | Cross-sectional study | Adolescents between the age of 12-15 years | To determine whether  socio economic inequalities are experienced amongst them are correlated to dental caries. | Dental caries were examined clinically by using DMFT method amongst three different socio economic groups in accordance with area  of residence from urban slums, middle-class areas and resettlement colonies. | Dental caries experience | The adolescents had higher odds of having dental caries in resettlement colonies and urban slums. |
| Kothia et al, 2015, India | Narrative Review | NA | To assess the National  Oral Health Policy in India | The literature search of 106 articles was performed both manually and electronically to yield a final of 34 articles. | NA | Highlighted the various policy changes including barriers, solutions and challenges. |
| Shah et al, 2003, Delhi | Cross-sectional study | Elderly | To assess gender disparities’ between elderly men and women with regard to oral diseases | A total of 524 rural and 716 urban elderly were studied utilizing the WHO Oral Health Screening Proforma to evaluate their oral health status and treatment needs. | Gender disparity in oral health and disease | Elderly Men were more likely to seek dental treatment due to higher education, SES, economic independency as compared to elderly women |
| Hallapa et al, 2014, India | Narrative Review | Dental health-workforce in India | To assess distribution of dental workforce, their related inequalities and their impact | SWOT Analysis of dental workforce. | NA | The article highlighted the need for dental workforce planning and implementation of strategies to facilitate better utilization of the available dental workforce and overcoming disparities both intrastate and interstate within India |
| Manpreet et al, 2021, Bangalore | Cross-sectional study | Transgenders and Controls | To evaluate oral health related parameters, amongst transgender adults, including Candida growth and intensity. | Two subgroups of adults including 40 transgenders and 40 controls were evaluated using the WHO oral health assessment form as well as samples were obtained to study Candida growth from the dorsum of the tongue. | Oral Health status and related oral mucosal disorders | Transgender adults reported poor oral health and many oral mucosal disorders which might be associated with a higher rate of behavioural risk factors such as tobacco and alcohol consumption. |
| Saha et al, 2014, India | Narrative Review | NA | To give an overview of existing  state of oral health services and their benefits, shortcomings and solutions | Review | NA | To make Oral Health care affordable, six strategic areas were highlighted in the paper including System infrastructure, Oral health data, Oral health promotion, Oral disease prevention, Dental manpower & Implementation and sustainability |
| Garcha et al, 2010, Pune | Cross-sectional study | Adults aged 35-45 years | To investigate and compare, amongst people from various social classes regarding their barriers to accessing oral health care. | Convenience sampling:250 people aged 35-45 years were selected with 50 participants each divided based on social classes. | Barriers to oral health care | Oral Health care services ,article depicts barriers with respect to to access both differences and similarities in social classes |
| Megalamanegowdru et al, 2013,  Karnataka,India | Narrative Review | NA | To assess SES and oral health  inequities | Review | SES and Oral Health Inequity | Lower SES is linked with poorer oral health. |
| Siddharthan et al, 2021, India | Narrative Review | NA | To assess oral health care  services in India | Review | NA | The article focuses on both barriers as well as possible solutions to provide better dental care services in India. |
| Singh et al, 2013, India | Narrative Review | NA | To address oral health  inequity, access concerns and workforce issues in India. | Review | NA | This paper highlights the policy and workforce related barriers and solutions towards oral health care services in India. |
| Purohit et al, 2022, India | Narrative Review | NA | To assess need for universal oral health coverage. | Review | NA | The article speaks about policy changes and shift towards prevention of dental disease. |
| Koyio et al, 2016, Hyderabad | Workshop Report-Original article | HIV patients | To assess oral health disparities and oral health promotion amongst individuals with HIV. | Oral health needs of individuals with HIV were discussed at a workshop to address the related inequalities at the global level and the strategies towards better research in this area. Dental students and dentists attending the 7th World Workshop on Oral Health & Disease in AIDS were the participants. | Oral health needs of HIV positive people | The paper discussed upstream and downstream measures for oral health promotion for individuals with HIV. Community oriented health programs, extensive and inclusive access to care which is evidence based and free from discrimination were identified as important strategies. |
| Kakde et al, 2013, India | Cross-sectional survey | Dentists | To evaluate the perceived challenges encountered by dental health professionals in India. | An online survey was administered amongst dentists practising in India and explored both quantitative and qualitative aspects. | Identify issues and challenges | Oral disease burden high among Indians. Review and update of dental education. Indian Diaspora can support dental care through mentoring, clinical expertise and charity. |
| Anil et al, 2019, Wayanad, Kerala | Program evaluation-  Retrospection of oral rehabilitative program | Tribes in Wayanad | To assess the oral rehabilitative  program –Amrithasmitham | The tribal outreach program has  been delivering free dentures since 2008. three day camp comprise 30-40 dental staff on average | Oral health | The article outlines an outreach program to the tribal community which can be replicated in unreachable locations or in geographically  reachable yet physically challenged geriatric population. |
| Rajput et al, 2020, Bangalore | Cross-sectional study | School chidren- differently-abled and healthy | To evaluate the oral health related inequalities among healthy and differently-abled school children. | Two groups of 300 each including differently-abled and healthy school children respectively were assessed for dental caries using DMFT index. | DMFT and SES | Mean DMFT higher in study group. Lower SES higher caries.Visually impaired children, , children suffering from polio, speech and hearing impaired children and children without caries in deciduous dentition had less chances to experience caries in permanent teeth. |
| Gupta et al, 2015, Haryana | Prospective cohort study | Automobile workers (Adults) | To assess the interrelationships between individual and environmental factors on subjective as well as clinical oral health outcomes in adults. | Self-reported measures of  OHQoL, individual and environmental factors were collected at baseline and 3-mo follow-up | Self-reported measures of  OHQoL, individual and environmental factors | Factors such as SES along with higher SOC, social support and less stress levels…are linked with better oral health quality of life. |
| Kadanakuppe et al, 2013, Karnataka, India | Cross-sectional study | Iruliga tribal community | To evaluate the status of oral health and treatment needs among Iruliga tribal community residing at Ramanagara District, Karnataka | World Health Organization Oral Health Assessment Form 1997 was used to study 2605 Iruligas. | Oral health status | Oral disease burden low in Iruligas indicating good oral hygiene via traditional oral hygiene methods but problem to accessing care |
| Mehta et al, 2015, India | Narrative Review | NA | To assess social determinants of  health and oral health in India | The papers selected following literature search across PubMed and Google Scholar were used to study the social determinants of health and oral health from different countries. While government websites and registries. Were used to obtain data relevant to Indian context. | NA | Health care system is the most  critical social determinant of health. Malnutrition increases burden of oral diseases.Inequalities in oral health mirror those in general health. |
| Mahal et al, 2006, India | Narrative Review | Dental health-workforce | To assess Implications of the growth of dental education in India | Data from freely available sources on official websites of the Indian Ministry of Health and via direct communication with individual institutions or their websites. | NA | Suggests using dental auxillaries for prevention of dental disease at low costs. |
| Kumar et al, 2021, Mumbai | Cross-sectional study | Dentists and dental students | To assess and compare the attitude of dental workforce in government and private dental schools in India towards Sexual and Gender Minorities (SGM) cohorts. | Medical Condition Regard Scale  (MCRS) was the study tool used to collect data | Dental workforce attitude  towards SGM | Mixed Responses towards  SGM indicates the need for altering the attitudes of dentists as well as spreading awareness about these gender minorities |
| Oberoi et al, 2017, Faridabad, Haryana | Cross-sectional study | Adult patients visiting dental OPD | To evaluate the effect of  socioeconomic status on the oral hygiene habits. | The questionnaire was administered which explored the demographic profile as well as the oral hygiene habits of the study population. | SES &Oral hygiene habits | Socioeconomic status had a huge influence on the oral hygiene practices of the participants. |
| Puzhankara et al, 2021, India | Narrative Review | NA | The models of medical-dental integration were reviewed, along with potential approaches for their incorporation into the healthcare landscape of India. | Literature was searched across Web of Science, PubMed/MEDLINE, CINAHL and Google Scholar for studies relevant to medical-dental integration. | NA | Oral health care equity can be realized by merging oral health services with overall healthcare. This inclusion can be accomplished by including periodontal and oral health monitoring within community-level surveillance of Non-Communicable Diseases. |
| Singh et al, 2011, Udupi,Karnataka | Cross-sectional study | 12 year old school children | To evaluate the oral health condition of 12-year-old students belonging to socially disadvantaged segments. | 2 groups- children from Ashram  schools & govt schools | Oral health status | Calculus, fluorosis, dental caries was higher in Ashram children who are socially disadvantaged. |
| Iyer et al, 2019, Bangalore | Cross-sectional study | Medical and dental officers | A situation analysis was carried out at PHCs to gain an understanding of resource availability and oral health seeking behavior, approached from the perspective of medical officers. | Questionnaire study | Knowledge and practices of medical and dnetal officers | Article highlights the need to give basic knowledge of oral health care to medical professionals as well as the need for provision of separate dental units within PHCs with adequate equipment |
| Mathur et al, 2015, India | Commentary | NA | The effectiveness of skill mix in dentistry for addressing inequalities in oral health were reviewed. | NA | NA | The dental auxillaries have the potential to decrease disparities in oral health by fulfilling essential treatment requirements and engaging in oral health promotion efforts within rural regions. |
| Bommireddy et al, 2016, Guntur, Andhra Pradesh | Cross-sectional study | Rural population aged 55 and more | To determine how oral health care is being used and recognize obstacles to its use within rural communities. | Stratified cluster sampling was done. Data collected using questionnaire | Dental service utilization | Among dentate elderly group, fear emerged as a frequently mentioned obstacle hindering the utilization of dental services. |
| Bhatt et al, 2017, Mangalore, Karnataka | Cross-sectional study | Fishermen aged 18-59 | To examine elements affecting the oral health condition and the utilization trends of oral health services within the fishing community. | A door-to-door survey was conducted involving 840 individuals. The assessment of oral health condition was carried out using the standard oral health survey form provided by the World Health Organization. | Dental service utilization | Poor dental care utilization.  The primary obstacle in seeking dental services was the absence of perceived necessity for oral health care. |
| Chandu et al, 2018, South India | Cross-sectional study | Students and staff of the six colleges  of a University | To gather information about the operations of satellite clinics and assess the effectiveness of an oral health promotion initiative within one of these satellite clinics. | Two-stage random sampling. The data collected through questionnaire | Dental service utilization | Poor dental care utilization was reported along with lack of awareness regarding available satellite clinic |
| Subramaniam & Muthukrishnan, 2021 - Chennai | Cross-sectional study | individuals with special needs | Recoginize barriers towards seeking dental care in people with special care needs | Questionnaire administration | Barriers towards accessing dental care. | Fear in general and fear of being injured in particular was the chief barrier among patients with special care needs. |
| Maheshwariet al, 2017, Rajasthan | Cross-sectional study | Schoolchildren | To create a communication tool for health risk targeting the oral health concerns of school children. | The methodology employed a one-step cluster sampling approach, involving a total of 920 participants for the assessment of their oral health condition. | Oral health staus | Variables linked with tooth decay included attendance at private schools, the age of the children, and inconsistent oral hygiene practices. |
| Satyarup et al, 2020, India | Narrative Review | NA | To assess Basic Package of Oral Care (BPOC) | Review | NA | Utilizing the current Primary Health Center staff, local assets, and working in conjunction with non-governmental organizations could offer the optimal solution for addressing the dental requirements of communities through the implementation of Basic Package of Oral Care. |
| Menon et al, 2016, Jalandhar, Punjab | Case study | Data collected from PHC and CHC | To assess provisioning of dental  health in public hospitals | The dental facilities and procedures conducted at the public hospital were assessed using a checklist based on the Indian Public Health Standards 2012 for Community Health Centers (CHCs) and district hospitals. Subsequently, differences within and between these public hospitals were examined and compared. | The dental facilities and dental procedures carried out at the public hospital were evaluated using a checklist. | In public hospitals, the dental outpatient department constituted around 5% to 10% of the total. The commonly conducted procedure was tooth extraction. Prosthodontic and orthodontic treatments were notably absent from the services provided by public hospitals. Additionally, dental clinics within Community Health Centers lacked dental auxiliaries, and there was an inadequacy of essential dental materials necessary for proper care. |
| Jawahar et al, 2022, Chennai | Cross-sectional study | Visually impaired adults | To evaluate the oral well-being and training requirements of individuals who have visual impairment. | A total of 207 patients with visual impairments who sought care at the specialized dental department of an Oral Medicine and Radiology unit within a private dental college & hospital and a private institute for the blind. | The procedures conducted at the public hospital were verified using a checklist. | The occurrence of dental cavities was substantial. There was a notable frequency of periodontal pocket and gingival bleeding, along with approximately 30% of the participants displaying fractures in their anterior teeth. The demand for dental treatments was significant among the visually impaired group, and a considerable proportion of those needs remained unaddressed. |
| Janakiram et al, 2018, India | Content analysis | NA | To analyze the level of political emphasis on oral health in India and to comprehend the underlying factors contributing to the political backing received by oral health initiatives. | The assessment was grounded in the political power framework initially formulated by Shiffman and Smith, and later adapted by Benzian et al. | NA | There seem to be a lack of unified and holistic strategy to tackle oral health issues. Limited use of dental services was observed. Additionally, there seems an absence of a nationwide monitoring system for oral well-being. |
| Chandrashekhar et al, 2011, Mysore | Cross-sectional study | Municipal employees | To evaluate the connection between socioeconomic factors and oral health status among municipal employees. | The necessary data was collected using the Oral Health Assessment form (1997) from the World Health Organization (WHO) and a pre-designed questionnaire. | Oral Health ststus | A reverse correlation existed between socioeconomic status and oral health condition, characterized by an elevated incidence of dental caries, as well as a greater prevalence of oral pre-malignant and malignant lesions among individuals belonging to lower SES. |
| Kumar et al, 2011, Udaipur | Cross-sectional study | 12-15 year old children | To contrast the oral health-related quality of life (OHRQoL) and the prevalence of dental caries between school children who reside with their parents and those who are orphans. | Within the participant pool of 536 children, 279 were accompanied by parents, while 257 were without parental guardianship. | Oral health related quality of life | Children in orphanages reported increased functional restrictions and diminished social well-being. |
| Radha et al, 2011, Bangalore | Cross-sectional study | Tibetans immigrants | To assess the connection between Acculturation and status of oral health among Tibetan immigrants in Bangalore. | Study sample-560. The modified Psychological-Behavioral Acculturation Scale was employed for the measurement of acculturation, and the recording of dental caries experience and periodontal status took place. | Oral health status and acculturation status | In the high acculturation category, 49.1% comprised females, and 50.95% were males. The occurrence of dental caries was nearly equivalent in both the high and low acculturation groups. Notably, individuals with lower acculturation and males exhibited greater prevalence of periodontal disease compared to those with higher acculturation and females. |
| Verma et al., 2012 - Chandigarh | Cross-sectional study | General Population as well as dentists working in public health set-ups in rural and urban areas. | Evaluate the access to public  dental care services and related factors in Chandigarh | Community survey and health  facility survey in rural and urban areas. | Various aspects that I nfluence access to dental care and common problems in government dental clinics. | People in rural areas prefer government dental set-ups while affordability of care was higher in urban areas. Perceived costs of dental treatments were found to be higher than actual costs of treatment. People from rural areas took longer to reach the dentist which could be due to the higher workload on dentists secondary to more number of patients or lack of assistance or both. |
| Kadaluru et al, 2012, Bangalore | Cross-sectional study | Adults attending community  outreach programs | To evaluate the utilization of dental care services in adults visiting outreach programs. | Adults aged 18–55 years.  Study tool- questionnaire | Dental service utilization | Utilization of dental services was poor with high cost cited as the main barrier. Tooth extraction and acute symptoms were reasons for visiting the dental clinic rather than for prevention. |
| Chathurvedi et al, 2013, Raipurani districtof India | Cross-sectional study | Schoolchildren | To assess the influence of proximity to dental schools on oral hygiene awareness as well as oral health status in school children. | Children between the ages of 9-11yr, 12-13 yrs and 14- 15 yrs from a sample size of 187 were assessed based on oral health assessment Performa. | Oral health status | Proximity to dental schools was associated with a better oral health status among school children. |
| Nagaraj et al, 2014, Rajasthan | Cross-sectional study | Rural Female population | To study the effect of cultural practices on the oral health status and oral health care service utilization. | Systematic random sampling. Data collected using questionnaire | Dental service utilization | There was prevalence of various myths concerning dental treatment and indigenous methods showed more prevalence among uneducated females as compared to educated females. These beliefs further prevented them from utilizing dental care services. |
| Gupta et al, 2014, Rajasthan | Cross-sectional study | Rural population | To evaluate the utilization of dental services among rural population. | Total of 5476 people between 20 to 60 years and more were evaluated using a questionnaire | Dental service utilization | Majority had had never visited a dentist. Pain was a major reason for the last dental visit. Feeling of  insecurity is significantly common among the elderly. Cost of dental treatment was the major barrier in Dental service utilization |
| Singh et al, 2015, India | Narrative Review | Geriatric population | To evaluate the oral health status of geriatric population in India | Review | NA | Oral health policy formulation for the geriatric patients is required where dental auxiliaries along with Primary Health Centres (PHCs) and Community Health Centres  (CHCs) could be employed to increase access to geriatric patients. |
| Gupta et al, 2015, Moradabad | Cross-sectional study using secondary data | Preschool children between 3-5 years of age. | To evaluate the prevalence of dental caries and the related treatment needs in pre-school hildren | The children were evaluated for dental caries and related treatment needs utilizing the modif ed WHO proforma 1997. | Oral health status and Treatment needs | The article highlights the need for oral health education of parents and teachers as almost 50% of this population suffered from caries. |
| Bhandari et al, 2015, Secondary data | Survey | 18 years or older adults | This paper investigated the association between  utilization of dental care services and income inequality. | Sixty-six World Health Survey countries were selected where data from 223,299 adults was studied. Gini coefficient was used to measure national level income inequality. .Use of dental services was the outcome measure | Income Inequality and utilization of dental care services | Income inequality as well as funding in health sector plays an important role in use of dental services. |
| Janakiram et al, 2017, India | Narrative Review | NA | To present a detailed study of oral health equity in India. | Review | NA | The article oulined various suggestions to achieve equity in oral health care in India. |
| Krishnan et al, 2019, Chennai | Cross-sectional study | Urban slum dwellers | Association between  sociodemographic factors and unmet dental needs. | 430 participants were studied  DMFT, TI, TNI recorded | Oral health status via DMFT, TI, TNI | Low utilization of services was reported. Income is the major detrimental factor in availing treatment |
| Shwetha et al., 2019 - Chikkaballapur  District, Karnataka | Cross-sectional study | Accredited social  health activists (ASHAs) | To develop a tool to assess the  attitude to raise awareness towards oral cancer in ASHAs | Attitude questionnaire was  administered to ASHAs | Attitude was assessed | Involving ASHAs in prevention of oral cancer will be beneficial for their communities as the prevalence of oral cancer is higher in rural areas due to tobacco use among low socioeconomic strata. |
| Suresh et al, 2021, Mangalore | Mixed Methods Study | Children with special needs | To assess the reasons for reduced utilization of oral health services among children with disabilities who attended a coordinated health related programme in Mangalore. | This study employed a mixed-methods study design to assess dental service utilisation quantitatively, and qualitatively study the barriers for the same. | Dental service utilization and the associated barriers. | Caregivers felt that cognitive barriers were the major reason for underutilisation of the oral health services in children with special needs. |
| Subramaniam et al, 2020, Bangalore | Cross-sectional study | 3-5 year old preschool children | To assess oral health related quality of life (OHRQoL) and dental caries among preschool children residing in rural and urban areas. | 1545 preschool children between 3-5 years of age from urban and rural Bangalore were evaluated for dental caries utilizing WHO criteria. Along with that OH-ECQOL proforma on OHRQoL was administered on the parents. | OHRQoL  Dental Caries Status | Oral health related quality of life was worse in preschool children belonging to rural Bangalore in comparison with those residing in urban Bangalore. |
| Kumar et al, 2010, Ambala, Haryana | Cross-sectional study | Adults aged 20-74 years | Rural population of Ambala were studied to evaluate their oral health status as well as treatment needs. | 1250 subjects between  20-74 years of age underwent an interview using a structured questionnaire along with examination using modified WHO format 1997. | Oral health status and Treatment  needs | Socioeconomic status, gender, education, & brushing frequency were found to be  associated with dental caries. Deleterious habits were more among uneducated and poor social class. |
| Jain 2013, Virajpet | Cross-sectional study | Adult Population  visiting CHC's and PHCs  in Virajpet | Explore Sociodemographic charecteristics, Dental behaviours and barriers for utilization of oral healthcare | Questionnaire study | Barriers in utilization of dental care | Need to take care of individual factors which act as bariiers i.e. knowledge, fear, transport, cost |
| Gambhir 2013, India | Narrative Review | NA | Review of papers pertaining to utilization of dental care in India. | Review | NA | Various barriers especially on the individual level pertaining to utilization of dental care were outlined in this study. |
| Tandon 2004, India | Commentary | NA | Outlines the challenges with respect to dental workforce in India | NA | NA | Geographrical imbalance in distribution of dentists, Lack of Dental Auxillaries, Insufficient specialist training and Migration of dentists were identified as reasons for inadequate dental workforce planning and development in India. |
| Sehgal 2011, India | Narrative Review | NA | Suggestions regarding public private partnership were presented | Review | NA | Public Private Partnership and dental insurance as a solution to reducing disparities in oral health care was presented in this article |
| Chavan 2012, India | Narrative Review | NA | Lists recommendations to establish Public Private Partnership for oral health | Review | NA | The article presented pros and cons of  Public Private Partnership for oral health promotion and care |
| Jawedkar 2013, India | Narrative Review | NA | To explore a new model for oral health promotion in children | Review | NA | The article proposed oral health promotion among children and infants by using existing models of health promotion in children living in deprived areas. The article also presented examples from other countries. |
| Vundavalli 2014, India | Historical Cross Section Study on various data sources | Various data sources | Discusses dental manpower planning in India | Discusses statistics behind dental workforce planning | Problems related to dental workforce planning. | This paper sheds light upon the shortcomings of the dental workforce planning in India, highlighting the need for equitable distribution of dentists across urban and rural areas as well as public and private sectors. |
| Balasubramaniam 2012, Guntur India | Cross Sectional Study | Dental students in different academic years | This study evaluated the attitudes of dental students in India towards substance misuse, HIV-positive status, intellectual disability lesbian, gay, bisexual, transgender (LGBT) orientation and acute mental illness. | Questionnaire with MCRS | Medical Condition Regard score | The dental students had highest regard for intellectual disability followed by acute mental illness, then lesser regard for HIV + patients and substance abuse. While they had the least regard for LGBT . |
| Samuel 2018, South India | Cross Sectional Study | High risk transgenders  and dental residents | This study examined the association between self- perceived barriers toward oral care, high risk transgender HIV status, and the willingness of the dentists to treat these patients during outreach community dental programs. | Questionnaire to assess self-perception of oral health as well as MCRS of dental residents | Questionnaire and medical condition regard score | Transgenders with high risk have poorer self perceived oral health and dental residents also have great fear of exposure to HIV |
| Paul 2014, Kolkata | Cross Sectional Study | People visiting tertiary  care hospital in Kolkatta | Evaluated the practices and awareness on oral hygiene while comparing the sociodemographic status of the patients. | A pre-tested pre-designed semi-structured schedule was used as the study tool. | Overall oral health practices were evaluated. | Males, Illiterates, low socioeconomic status and rural residents have poorer oral hygiene. The article suggests raising awareness among the vulnerable groups |
| Khemka 2015, India | Narrative Review | NA | Ways to increase accessibility to dental care | Review | NA | The paper lists various methods to increase accessibility to dental care services in India. |
| Tandon 2012, Karnataka | Cross Sectional Study | Rural Population | This study evaluated the use of a Mobile Dental Clinic while providing dental services to the rural population. | Pre and Post intervention i.e. Oral health education program, assessment of parameters to knowledge, attitude, practice and satisfaction in two groups was undertaken during the study. | Parameters to assess Knowledge, satisfaction attitude and practice. | Mobile dental vans were well accepted by rural people and the people were enthusiastic and it motivated them towards better oral health. Improvement in oral health parameters post mobile dental van education and treatment. |
| Sandhu 2014, India | Content Analysis/Survey- State by state analysis on secondary data | Dental Schools in India | State‐by‐state analysis of dental school distribution and comparison with populations to enhance our understanding of dental education in India | State by state analysis of dental schools in India | Distribution of dental colleges | The article sheds light upon maldistribution of dental schools across states in India. The paper also highlights that most of the population in majority states is located at a distance of more than 40 kms from dental schools. |
| Chandu 2017, India | Narrative Review | NA | To provide different ways to enhance accessibility to dental care in rural India. | Review | NA | Various strategies for increasing access to dental care in India have been listed in this article |
| Gambhir 2016, India | Short communication | NA | The paper highlights important aspects while formulating National Oral Health Policy. | Review of the situation | NA | Recommendations for modifications in national oral health policy are discussed by the authors. |
| Yadav 2014, India | Narrative Review | NA | The paper explores the inequity in oral health from various angles. | Review | NA | Focusses on the addressal of various factors contributing to oral health inequity. |
| Kishor 2010, India | Narrative Review | NA | Summation of reasons behind inequalities in oral health care | Review | NA | Discusses verious factors including individual factors, barriers due to dental education, workforce planning and faulty policies and lack of organised public health services in oral health care in India. |
| Adayanathaya 2017, Kerala | Cross-sectional study | Dental Practitioners  regarding chidren with  special care needs | Barriers faced by dental practitioners for treating children with special care needs | Questionnaire study | Hurdles in treatment | Level of training and motivation of caretakers were the main barriers to treating children with special care needs |
