# Supplementary material for: A scoping review exploring oral health inequalities in India: a call for action to reform policy, practice and research
Source: Int J Equity Health. 2023 Nov 21;22:242. doi: 10.1186/s12939-023-02056-5 (PMC10664303; doi:10.1186/s12939-023-02056-5)
Supplement: Supplementary file 3 — Supplementary Material 3 [file 12939_2023_2056_MOESM3_ESM.docx]

| **Author(s), year of publication** | **Preliminaries** | **Introduction** | **Design** | **Sampling** | **Data Collection** | **Ethical** | **Results** | **Discussion** | **Total** | **Total %** | **Quality of the study** |
| --- | --- | --- | --- | --- | --- | --- | --- | --- | --- | --- | --- |
| Kailembo et al, 2018 | 4 | 5 | 5 | 5 | 4 | 5 | 5 | 5 | 38 | 95 | High |
| Mathur et al, 2016 | 4 | 4 | 4 | 4 | 4 | 5 | 5 | 3 | 33 | 82.5 | Medium |
| Mathur et al, 2014 | 4 | 3 | 4 | 5 | 4 | 5 | 5 | 4 | 34 | 85 | Medium |
| Kothia et al, 2015 | 3 | 2 | 3 | 3 | 3 | 5 | 2 | 2 | 23 | 57.5 | Low |
| Shah et al, 2003 | 3 | 4 | 5 | 5 | 5 | 4 | 5 | 3 | 34 | 85 | Medium |
| Hallapa et al, 2014 | 4 | 2 | 2 | 2 | 2 | 5 | 3 | 3 | 23 | 57.5 | Low |
| Manpreet et al, 2021 | 5 | 4 | 5 | 3 | 5 | 5 | 5 | 5 | 37 | 92.5 | High |
| Saha et al, 2014 | 2 | 2 | 0 | 0 | 0 | 5 | 2 | 2 | 13 | 32.5 | Low |
| Garcha et al, 2010 | 4 | 4 | 4 | 3 | 3 | 3 | 4 | 4 | 29 | 72.5 | Medium |
| Megalamanegowdru et al, 2013 | 1 | 1 | 0 | 0 | 0 | 5 | 2 | 2 | 11 | 27.5 | Low |
| Siddharthan et al, 2021 | 3 | 2 | 0 | 0 | 0 | 4 | 1 | 2 | 12 | 30 | Low |
| Singh et al, 2013 | 3 | 2 | 0 | 0 | 0 | 4 | 1 | 3 | 13 | 32.5 | Low |
| Purohit et al, 2022 | 4 | 3 | 0 | 0 | 0 | 5 | 3 | 4 | 19 | 47.5 | Low |
| Koyio et al, 2016 | 4 | 4 | 3 | 0 | 3 | 0 | 4 | 4 | 22 | 55 | Low |
| Kakde et al, 2013 | 4 | 4 | 4 | 4 | 4 | 5 | 4 | 5 | 34 | 85 | Medium |
| Anil et al, 2019 | 4 | 4 | 4 | 3 | 3 | 5 | 3 | 3 | 29 | 72.5 | Medium |
| Rajput et al, 2020 | 5 | 5 | 5 | 4 | 5 | 5 | 5 | 5 | 39 | 97.5 | High |
| Gupta et al, 2015 | 4 | 4 | 4 | 5 | 5 | 5 | 5 | 5 | 37 | 92.5 | High |
| Kadanakuppe et al | 3 | 3 | 3 | 3 | 4 | 3 | 4 | 3 | 26 | 65 | Medium |
| Mehta et al, 2015 | 3 | 3 | 3 | 3 | 3 | 5 | 3 | 3 | 26 | 65 | Medium |
| Mahal et al, 2006 | 4 | 4 | 4 | 3 | 4 | 5 | 3 | 4 | 31 | 77.5 | Medium |
| Kumar et al, 2021 | 5 | 5 | 5 | 5 | 5 | 5 | 5 | 5 | 40 | 100 | High |
| Oberoi et al, 2017 | 4 | 4 | 3 | 3 | 3 | 5 | 5 | 4 | 31 | 77.5 | Medium |
| Puzhankara et al, 2021 | 4 | 4 | 1 | 1 | 1 | 5 | 3 | 4 | 23 | 57.5 | Low |
| Singh et al, 2011 | 4 | 4 | 4 | 4 | 4 | 4 | 3 | 4 | 31 | 77.5 | Medium |
| Iyer et al, 2019 | 4 | 4 | 3 | 4 | 3 | 1 | 3 | 3 | 25 | 62.5 | Medium |
| Mathur et al, 2015 | 4 | 4 | 3 | 3 | 3 | 5 | 3 | 3 | 28 | 70 | Medium |
| Bommireddy et al, 2016 | 4 | 4 | 4 | 4 | 3 | 0 | 4 | 4 | 27 | 67.5 | Medium |
| Bhatt et al, 2017 | 4 | 4 | 4 | 4 | 3 | 5 | 5 | 4 | 33 | 82.5 | Medium |
| Chandu et al, 2018 | 4 | 3 | 4 | 4 | 3 | 5 | 4 | 4 | 31 | 77.5 | Medium |
| Subramaniam & Muthukrishnan, 2021 | 4 | 4 | 4 | 2 | 3 | 1 | 3 | 3 | 24 | 60 | Low |
| Maheshwariet al, 2017 | 3 | 3 | 3 | 4 | 3 | 4 | 4 | 3 | 27 | 67.5 | Medium |
| Satyarup et al, 2020 | 2 | 2 | 0 | 0 | 0 | 5 | 2 | 3 | 14 | 35 | High |
| Menon et al, 2016 | 4 | 3 | 3 | 3 | 3 | 3 | 3 | 4 | 26 | 65 | Medium |
| Jawahar et al, 2022 | 4 | 4 | 4 | 4 | 4 | 5 | 4 | 5 | 34 | 85 | Medium |
| Janakiram et al, 2018 | 4 | 4 | 3 | 2 | 3 | 4 | 4 | 5 | 29 | 72.5 | Medium |
| Chandrashekhar et al, 2011 | 4 | 3 | 4 | 4 | 4 | 4 | 4 | 4 | 31 | 77.5 | Medium |
| Kumar et al, 2011 | 4 | 3 | 4 | 3 | 3 | 4 | 4 | 3 | 28 | 70 | Medium |
| Radha et al, 2011 | 4 | 3 | 3 | 4 | 3 | 3 | 4 | 4 | 28 | 70 | Medium |
| Verma et al., 2012 | 3 | 3 | 4 | 4 | 3 | 0 | 4 | 3 | 24 | 60 | Medium |
| Kadaluru et al, 2012 | 3 | 3 | 4 | 3 | 4 | 5 | 4 | 4 | 30 | 75 | Medium |
| Chathurvedi et al, 2013 | 4 | 3 | 3 | 3 | 3 | 5 | 5 | 3 | 29 | 72.5 | Medium |
| Nagaraj et al, 2014 | 4 | 4 | 4 | 4 | 3 | 5 | 4 | 4 | 32 | 80 | Medium |
| Gupta et al, 2014 | 4 | 4 | 3 | 2 | 4 | 3 | 4 | 3 | 27 | 67.5 | Medium |
| Singh et al, 2015 | 3 | 3 | 0 | 0 | 0 | 5 | 2 | 3 | 16 | 40 | Low |
| Gupta et al, 2015 | 4 | 3 | 3 | 4 | 4 | 4 | 4 | 3 | 29 | 72.5 | Medium |
| Bhandari et al, 2015 | 4 | 4 | 4 | 4 | 4 | 4 | 5 | 4 | 33 | 82.5 | Medium |
| Janakiram et al, 2017 | 3 | 3 | 0 | 0 | 0 | 5 | 3 | 3 | 17 | 42.5 | Low |
| Krishnan et al, 2019 | 3 | 3 | 4 | 3 | 4 | 5 | 4 | 3 | 29 | 72.5 | Medium |
| Shwetha et al., 2019 | 4 | 4 | 5 | 4 | 5 | 5 | 5 | 3 | 35 | 87.5 | High |
| Suresh et al, 2021 | 5 | 5 | 4 | 3 | 4 | 5 | 5 | 5 | 36 | 90 | High |
| Subramaniam et al, 2020 | 4 | 4 | 4 | 4 | 4 | 4 | 5 | 4 | 33 | 82.5 | Medium |
| Kumar et al, 2010 | 3 | 3 | 4 | 4 | 4 | 3 | 4 | 3 | 28 | 70 | Medium |
| Jain 2013 | 3 | 3 | 3 | 3 | 3 | 4 | 4 | 4 | 27 | 67.5 | Medium |
| Gambhir 2013 | 3 | 3 | 0 | 0 | 0 | 5 | 3 | 3 | 17 | 42.5 | Low |
| Tandon 2004 | 3 | 3 | 0 | 0 | 0 | 5 | 4 | 4 | 19 | 47.5 | Low |
| Sehgal 2011 | 0 | 3 | 0 | 0 | 0 | 5 | 3 | 3 | 14 | 35 | Low |
| Chavan 2012 | 3 | 4 | 0 | 0 | 0 | 5 | 4 | 4 | 20 | 50 | Low |
| Jawedkar 2013 | 4 | 4 | 0 | 0 | 0 | 5 | 4 | 4 | 21 | 52.5 | Low |
| Vundavalli 2014 | 3 | 4 | 4 | 0 | 4 | 5 | 4 | 5 | 29 | 72.5 | Medium |
| Balasubramaniam 2012 | 4 | 4 | 4 | 3 | 3 | 5 | 4 | 5 | 32 | 80 | Medium |
| Samuel 2018 | 5 | 4 | 4 | 3 | 4 | 5 | 4 | 4 | 33 | 82.5 | Medium |
| Paul 2014 | 4 | 4 | 3 | 2 | 3 | 4 | 4 | 3 | 27 | 67.5 | Medium |
| Khemka 2015 | 3 | 4 | 0 | 0 | 0 | 5 | 4 | 4 | 20 | 50 | Low |
| Tandon 2012 | 4 | 5 | 4 | 4 | 4 | 5 | 4 | 4 | 34 | 85 | Medium |
| Sandhu 2014 | 4 | 4 | 3 | 2 | 3 | 5 | 4 | 3 | 28 | 70 | Medium |
| Chandu 2017 | 4 | 4 | 0 | 0 | 0 | 5 | 4 | 4 | 21 | 52.5 | Low |
| Gambhir 2016 | 4 | 4 | 3 | 2 | 2 | 5 | 3 | 3 | 26 | 65 | Medium |
| Yadav 2014 | 3 | 3 | 0 | 0 | 0 | 5 | 3 | 3 | 17 | 42.5 | Low |
| Kishor 2010 | 3 | 3 | 0 | 0 | 0 | 5 | 3 | 3 | 17 | 42.5 | Low |
| Adayanathaya 2017 | 3 | 4 | 3 | 2 | 2 | 3 | 4 | 4 | 25 | 62.5 | Medium |
